# Evaluating Glycemic Control in Patients of South Asian Origin With Type 2 Diabetes Using a Digital Therapeutic Platform: Analysis of Real-World Data

**DOI:** 10.2196/17908

**Published:** 2021-03-25

**Authors:** Arjun Krishnakumar, Ritika Verma, Rajeev Chawla, Aravind Sosale, Banshi Saboo, Shilpa Joshi, Maaz Shaikh, Abhishek Shah, Siddhesh Kolwankar, Vinod Mattoo

**Affiliations:** 1 Wellthy Therapeutics Pvt Ltd Mumbai India; 2 Department of Diabetology, North Delhi Diabetes Centre New Delhi India; 3 Department of Diabetology, Diacon Hospital Bengaluru India; 4 Department of Diabetology, Dia Care-Diabetes Care and Hormone Clinic Ahmedabad India

**Keywords:** digital therapeutics, type 2 diabetes, behavior change, diabetes self-management, lifestyle intervention, mobile phone

## Abstract

**Background:**

Digital therapeutics are evidence-based therapeutic interventions driven by high-quality software programs for the treatment, prevention, or management of a medical disorder or disease. Many studies in the western population have shown the effectiveness of mobile app–based digital therapeutics for improving glycemic control in patients with type 2 diabetes (T2D). However, few studies have assessed similar outcomes in the South Asian population.

**Objective:**

This study aims to investigate the real-world effectiveness of the Wellthy CARE digital therapeutic for improving glycemic control among the South Asian population of Indian origin.

**Methods:**

We analyzed deidentified data from 102 patients with T2D from India enrolled in a 16-week structured self-management program delivered using the Wellthy CARE mobile app. Patients recorded their meals, weight, physical activity, and blood sugar in the app, and they received lessons on self-care behaviors (healthy eating, being active, monitoring, medication adherence, problem solving, healthy coping, and reducing risks); feedback provided by an artificial intelligence–powered chatbot; and periodic interactions with certified diabetes educators via voice calls and chats. The primary outcome of the program was a change in glycated hemoglobin A_1c_ (HbA_1c_). Secondary outcomes included the difference between preintervention and postintervention fasting blood glucose (FBG) and postprandial blood glucose (PPBG) levels; changes in BMI and weight at the completion of 16 weeks; and the association between program engagement and the changes in HbA_1c_, FBG, and PPBG levels.

**Results:**

At the end of 16 weeks, the average change in HbA_1c_ was –0.49% (n=102; 95% CI −0.73 to 0.25; *P*<.001). Of all the patients, 63.7% (65/102) had improved HbA_1c_ levels, with a mean change of −1.16% (n=65; 95% CI −1.40 to −0.92; *P*<.001). The mean preintervention and postintervention FBG levels were 145 mg/dL (n=51; 95% CI 135-155) and 134 mg/dL (n=51; 95% CI 122-146; *P*=.02) and PPBG levels were 188 mg/dL (n=51; 95% CI 172-203) and 166 mg/dL (n=51; 95% CI 153-180; *P*=.03), respectively. The mean changes in BMI and weight were –0.47 kg/m^2^ (n=59; 95% CI −0.22 to −0.71; *P*<.001) and –1.32 kg (n=59; 95% CI −0.63 to −2.01; *P*<.001), respectively. There was a stepwise decrease in HbA_1c_, FBG, and PPBG levels as the program engagement increased. Patients in the highest tertile of program engagement had a significantly higher reduction in HbA_1c_ (−0.84% vs −0.06%; *P*=.02), FBG (−21.4 mg/dL vs −0.18 mg/dL; *P*=.02), and PPBG levels (−22.03 mg/dL vs 2.35 mg/dL; *P*=.002) than those in the lowest tertile.

**Conclusions:**

The use of the Wellthy CARE digital therapeutic for patients with T2D showed a significant reduction in the levels of HbA_1c_, FBG, and PPBG after 16 weeks. A higher level of participation showed improved glycemic control, suggesting the potential of the Wellthy CARE platform for better management of the disease.

## Introduction

### Background

South Asians represent approximately one-fourth of the global population and are at a disproportionately higher risk of being diagnosed with type 2 diabetes (T2D). The global population of patients with diabetes was estimated to be 463 million in 2019 and is projected to reach 578 million by 2030, while that in South East Asia was 88 million in 2019 and is expected to reach 115 million by 2030 [1]. This alarming rise has been attributed to the Asian Indian phenotype and lifestyle changes associated with urbanization and sedentary living [2]. Many studies have reported poor glycemic control in patients with T2D in South Asian countries. [3-5]. In India, the management of T2D is limited by the high burden and early onset of the disease [6]. The Asian phenotype with diabetes has shown marked differences in terms of chemical and biochemical characteristics, such as more significant beta cell dysfunction, higher abdominal adiposity, and a higher susceptibility to developing cardiovascular complications than the Caucasian population [7]. Compared with the general population, diabetes, which is implicated as one of the principal causes of premature heart attacks and death, occurs at 50% higher rates and approximately 5 to 10 years earlier in South Asian patients. Moreover, only a quarter of all South Asian patients achieve their key clinical targets compared with the Caucasian population [5].

Lifestyle management is a fundamental aspect of diabetes care and includes diabetes self-management education and support, medical nutrition therapy, physical activity, smoking cessation counseling, and psychosocial care. Behavioral and lifestyle interventions have been recognized as integral aspects of improving outcomes in these patients. An integrated self-management regimen requires patients to adhere to regular self-glucose monitoring, healthy eating, exercise, and regular physician and specialist visits [8]. Self-monitoring of dietary calorie and fat intake and physical activity are also key strategies [9].

Despite robust evidence that lifestyle interventions in diabetes lead to a reduction in risk factors for cardiovascular diseases [10], self-care behaviors for the management of diabetes among South Asians remain poor because of the lack of knowledge, awareness, and education [11,12]. Moreover, patients solely depend on physicians as a source of information and disease knowledge, which they are unable to provide, as they often lack the time needed to effectively engage patients in self-management behaviors during and between consultation visits [13]. Poor glycemic control leads to earlier incidence and greater severity of diabetes-associated complications, leading to higher morbidity, poor quality of life, and loss of productivity, resulting in increased financial burden [14].

Studies that have examined the effectiveness of diabetes education programs in improving glycemic control in Asian Indians have reported contrasting results depending on the region and intervention [15,16]. One possible explanation for the differences in the results is the cultural appropriateness of these programs. South Asian Indians are a diverse culture with numerous languages and dialects, castes and clans, and cooking styles; therefore, it is imperative to understand the influence of misperceptions, culture, and values on disease management in this population, especially because implementation of culturally appropriate programs leads to a greater reduction in hemoglobin A_1c_ (HbA_1c_) levels [17]. Traditionally, diabetes education and support have been associated with hospital- or clinic-based practices. However, modern care practices aim to meet these needs through the use of digital interventions that combine monitoring, continuous reinforcement of behavior modification, and personalized therapy using technology [13,18,19].

The widespread adoption of mobile phone technologies in middle- and low-income countries underscores the potential for digital therapeutics to negotiate and overcome the practical roadblocks inherent to conventional physicians and in-clinic–based (eg, face-to-face) interventions and education [18]. Technology-enhanced interventions are cost-effective to enable population management and specialized care accessible to adults with T2D in real time across distant geographies [20-22]. However, little is known about the effect of digital therapeutics in South Asian populations. Therefore, there is a need for approaches that use culturally adapted digital therapeutic interventions specifically for this population [23].

### Objectives

On the basis of the role of emerging evidence, we hypothesized that a digital therapeutic with an artificial intelligence (AI)–powered decision support system could enhance multiple behavior patterns (self-monitoring of diet, exercise, weight, and blood glucose) among South Asian patients with T2D. The aim of this study is to assess the real-world effect of the Wellthy CARE digital therapeutics platform on glycemic control (HbA_1c_ and blood sugar levels) and other health outcomes (weight) in patients with T2D after 16 weeks of the program.

## Methods

### Research Design and Participants

We conducted an analysis of deidentified data for 102 patients with T2D on the Wellthy CARE mobile app. The analysis involved preintervention and postintervention assessment of HbA_1c_, fasting blood glucose (FBG), postprandial blood glucose (PPBG), BMI, and weight and program engagement measures in a convenience sample of adults with a self-reported diagnosis of T2D.

Participants were recruited through 2 channels: (1) a network of primary care clinics and diabetes centers through referrals from their treating physicians and (2) a social media campaign seeking participants for a smartphone-based diabetes management program. Patients expressed interest in participating in the program either by (1) calling a telephone number provided on the program recruitment handout or (2) filling out a preregistration form linked to the social media campaign where they voluntarily provided their mobile numbers. Patients who expressed interest in participating in the program received a screening phone call from the program staff members, explaining the program. Those who confirmed their willingness to participate underwent a screening interview (based on inclusion and exclusion criteria) to assess their eligibility. Preexisting T2D status was based on a combination of a self-reported diagnosis and a preintervention HbA_1c_ level of 6.5% or higher. User information was recorded on a web-based form, based on which the participants received a final decision on their eligibility to enroll.

Eligible patients were invited to download the mobile app from the Google Play Store using a unique link sent to them via SMS or text messages. On the mobile app, participants were then instructed to set up their unique account with their phone number and a one-time password sent to them. After the account set up, patients were requested to provide informed consent for their participation in the program and the use of their deidentified data for clinical research purposes.

The inclusion criteria for enrollment were as follows:

- Male or female adult patients aged 18 years or above at the time of enrollment, with a self-reported diagnosis of T2D (HbA_1c_ >6.5%)
- In possession of a personal Android (Google) smartphone with an active internet connection and the person should be comfortable in reading English content on the phone
- No history of a major surgical procedure during the previous 6 months or plan for any major surgical procedure in the next 6 months
- Absence of any medical condition that prevented them from walking for 15 minutes to 30 minutes a day

The exclusion criteria for enrollment were as follows:

- Diagnosis of type 1 diabetes, gestational diabetes mellitus, maturity-onset diabetes of young, or any other forms of diabetes
- Undergoing hemodialysis for chronic kidney disease
- History of any serious heart-related event such as a heart attack or stroke in the past 1 year
- Pregnant, nursing, or planning for a pregnancy in the next 6 months

Participants were not compensated for participation but were enrolled in the program at no cost. Informed consent for using their deidentified data for clinical research was obtained from each participant before enrollment in the program. Participation in the program was voluntary, and refusal to grant consent for the use of their deidentified data for research did not affect the participants’ enrollment in the program or the quality of care administered to them. This work involved only secondary analyses of deidentified data; hence, no ethics clearance was obtained. The program did not use any investigational product, and all physical assessments and education were used as usual customary care. No change in treatment was made, and patient care was provided according to the normal clinical standards.

### Program

The program was a 16-week structured lifestyle coaching delivered through the Wellthy CARE digital therapeutic consisting of the Wellthy CARE mobile app for Android smartphones, a web portal for health coaches to visualize patient data and communicate with them, and an AI-powered decision support system enabled across the platform (Multimedia Appendix 1). The Wellthy CARE app consisted of a secure messaging center and personal health record files with additional diabetes-related information (eg, laboratory values and details of treating physician) and provided access to a learning library and lesson plans and a logbook to review historical data. The comprehensive lesson plan for the program was based on the American Association of Diabetes Educator’s AADE7 self-care behaviors that encouraged patients to acquire skills for better diabetes self-management. The program coached participants along 7 tracks that covered eating healthy, becoming more active, improving self-monitoring, improving medication adherence, problem solving, reducing risk, and healthy coping. The Wellthy CARE app allowed patients to log diabetes self-care data (blood glucose, meals, physical activity, and weight) and diabetes management information such as laboratory reports on a mobile phone. The patient then received automated feedback delivered in real time through a *conversation* experience by an AI-powered chatbot that provided educational, behavioral, and motivational messaging specific to the data entered and in the context of the patient’s previous clinical, lifestyle, and behavioral data.

The program adopted a digital persuasion model that focused on improving the patients’ motivation, reducing the difficulty in performing a particular task, and then delivering appropriate triggers to the patients to take action. This was done through short culturally relevant content delivered in multiple formats such as informative lessons, videos, and protips along with quizzes and storyboards to reinforce the information and simple tasks to prompt actions. Patients could optionally enroll for challenges that required them to repeat an action multiple times during a specific period to enable skill formation. The program adopted a gamified approach to building skills and rewarded patients with virtual trophies as they completed the lessons, tasks, and challenges. A secure messaging system on the Wellthy CARE app was used by patients to communicate directly with their personal health coach at their own convenience.

Health coaches were *virtual* diabetes educators who regularly reviewed patient data, providing personalized feedback during each interaction, and also responded to patient queries. All patients received a voice call at the start of the program. Health coaches could supplement automated messages with electronic messages sent to patients via a secure messaging system. Health coach messages were based on longitudinal data trends, providing weekly and monthly summaries to patients on their performance. About 50% of patients made voice calls to or received voice calls from the health coach during the program, in addition to communicating over the messaging system. This was an incremental and supportive program for the existing standard of care recommended by the treating physician.

### Measures

A total of 102 participants completed and logged data during the 16 weeks on the Wellthy CARE platform. The participants self-reported their age, gender, height, and weight in the Wellthy CARE mobile app. All patients received a Gluco One (Dr Morepen) glucose meter and strips. The HbA_1c_ test was performed before and after the completion of the intervention by an independent pathological laboratory that reported the values directly.

### Outcomes

The primary outcome of this study was the change in HbA_1c_ level at the completion of the program (16 weeks). Secondary objectives included the difference between the mean preintervention and postintervention FBG and PPBG levels in participants who reported more than one blood sugar reading; change in BMI and weight in participants that reported more than one weight log; and association between program engagement, measured as the total number of interactions with the health coach and the AI-powered chatbot, and the change in HbA_1c_, FBG, and PPBG levels. In addition, we assessed the differences among patients who had an improvement of ≥0.4% in HbA_1c_ (responders) and those who did not (nonresponders).

### Statistical Analysis

Statistical analysis was performed using R software (version 3.4.3; the R Foundation). The results were analyzed to identify program starters (ie, those who completed at least one skill) and program completers (ie, those who completed at least 6 weeks of lessons or continued with health coaching through the duration of the program). Baseline characteristics were compared between subgroups using chi-square tests or Fisher exact test for categorical variables and 2-sample two-tailed *t* tests analysis for continuous variables and paired *t* tests for comparing preintervention and postintervention values for continuous variables. *P* values <.05 were considered statistically significant.

## Results

### Baseline Characteristics of Patients

**Figure 1:**
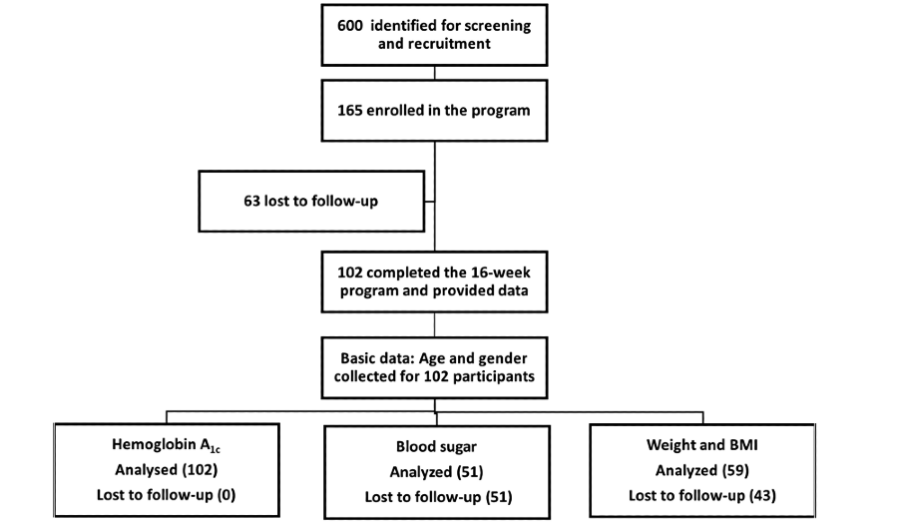
Participant recruitment and retention flowchart.

Figure 1 shows the data for participation and retention during the 16-week program. A total of 102 patients from 18 cities in India participated in the study and completed the program. The baseline characteristics of the participants are summarized in Table 1. There were no statistically significant differences in the baseline characteristics between male and female participants. The average duration of time spent with the personal health coach was 106 minutes (n=87; 95% CI 65-147) over the 16-week period, whereas that with the AI-powered chatbot was 88 minutes (n=102; 95% CI 66-110).

**Table 1:** Patient characteristics at the start of the program. All CI values are at 95% significance level. *P* value has been shown for comparison of male and female data.

Characteristics assessed	Overall	Male patients	Female patients	*P* value
Gender, n (%)	102 (100)	70 (68.6)	32 (31.4)	—^a^
Age (years), mean (CI)	50.8 (49.2-52.4)	51.4 (49.40-53.4)	49.7 (46.8-52.5)	.32
Weight (kg), mean (CI)	77.3 (80.5-74.0)	78.4 (82.2-74.7)	74.6 (81.1-68.1)	.31
BMI (kg/m^2^), mean (CI)	28.1 (29.2-26.4)	27.4 (28.7-26.1)	29.7 (31.8-27.6)	.07
Baseline hemoglobin A_1c_ (%), mean (CI)	8.5 (8.2-8.8)	8.5 (8.8-8.2)	8.6 (9.1-8.0)	.71

^a^Statistical comparison not applicable.

### Change in HbA_1c_

**Figure 2:**
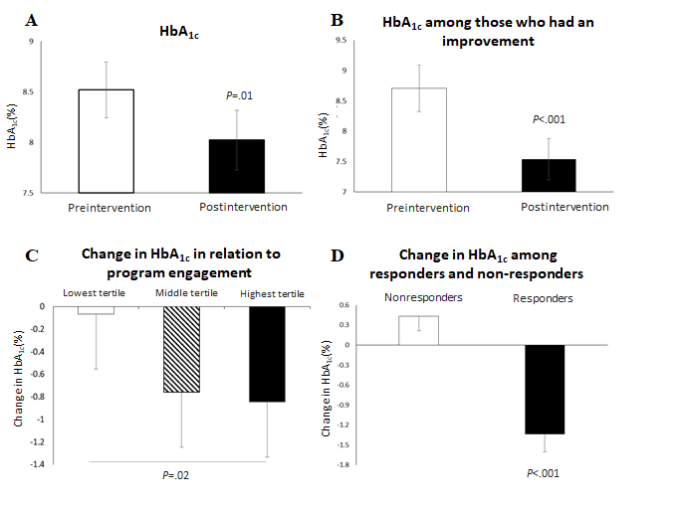
Comparison of HbA_1c_ levels. (A) Mean change in HbA_1c_ among all participants, (B) mean change in HbA_1c_ among those who had improvements in HbA_1c_ levels, (C) relationship between program engagement and change in HbA_1c_ levels, and (D) mean change in HbA_1c_ among responders and nonresponders. Error bars represent confidence intervals. HbA_1c_: hemoglobin A_1c_.

The mean change in HbA_1c_ among all patients was −0.49% (n=102; 95% CI −0.73 to −0.25; *P*<.001; Figure 2). Of all the patients, 63.7% (65/102) had improved HbA_1c_ levels with a mean change of −1.16% (95% CI −1.40 to −0.92; *P*<.001; Figure 2). Among the patients that had an improvement in HbA_1c_ levels, 48.0% (49/102) had a decrease of 0.5% or more and 27.5% (28/102) had a decrease of 1% or more; moreover, 29.4% (30) of the patients had a follow-up HbA_1c_ level less than or equal to 7%. Among those with a baseline HbA_1c_ level ≥7.5%, the mean change was −0.57% (n=59; 95% CI −0.90 to −0.25; *P*<.001).

The relationship between program engagement and changes in HbA_1c_ levels was assessed after 16 weeks of the program. There was a stepwise decrease in HbA_1c_ levels as the program engagement level increased (Figure 2). Among those with a baseline HbA_1c_>7.5%, the lowest tertile of mobile app engagers reduced their HbA_1c_ levels by 0.06% (95% CI −0.56 to 0.43) and the middle tertile of program engagers reduced their HbA_1c_ levels by 0.76% (95% CI −1.33 to −0.18); however, those in the highest tertile of program engagers significantly reduced their HbA_1c_ levels by 0.84% as compared with patients in lowest tertile (95% CI –1.33 to –0.35; lowest vs highest tertile, *P*=.02).

Responders were grouped as patients who showed an improvement of ≥0.4% in HbA_1c_ levels and those who did not were grouped as nonresponders. The mean change in HbA_1c_ among responders (51/102, 50%) was −1.34% (95% CI −1.07% to −1.60%), and that among nonresponders (51/102, 50%) was 0.43% (95% CI 0.65%-0.21%; *P*<.001; Figure 2).

### Change in Blood Sugar

**Figure 3:**
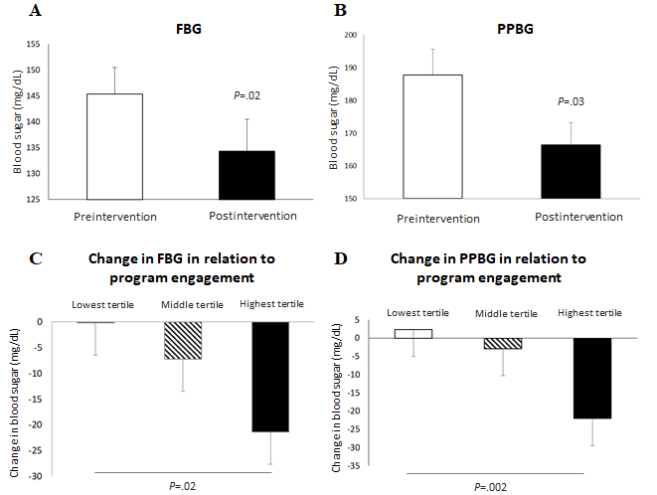
Change in blood sugar levels. Difference between (A) mean FBG levels and (B) mean PPBG levels reported in the first week and final week of the intervention. Relationship between mobile app use and change in (C) FBG and (D) PPBG levels. Error bars represent confidence intervals. FBG: fasting blood glucose; PPBG: postprandial blood glucose.

Among patients who reported more than one blood sugar reading (51/102), there was a significant difference between the mean preintervention and postintervention FBG (145 mg/dL; 95% CI 135-155 vs 134 mg/dL; 95% CI 122-146; *P*=.02) and PPBG (188 mg/dL; 95% CI 172-203 vs 166 mg/dL; 95% CI 153-180; *P*=.03) values (Figure 3).

There was a stepwise decrease in FBG and PPBG levels as the mobile app engagement level increased. The lowest tertile of app engagers reduced their FBG by 0.18 mg/dL and there was an increase in PPBG by 2.35 mg/dL; the middle tertile of app engagers reduced their FBG and PPBG by 7.25 mg/dL and 2.84 mg/dL, respectively; and those in the highest tertile of app engagers reduced their FBG by 21.4 mg/dL (*P*=.02, highest vs lowest) and PPBG by 22.03 mg/dL (*P*=.02, highest vs middle; *P*=.002, highest vs lowest; Figure 3).

**Figure 4:**
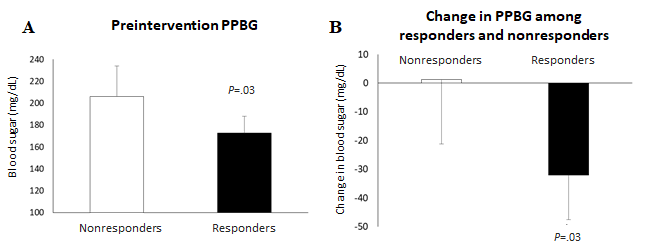
(A) Difference in the mean preintervention PPBG between nonresponders and responders. (B) Difference in the mean change in PPBG between the nonresponders and responders. PPBG: postprandial blood glucose.

Responders had a significantly lower mean preintervention PPBG of 172 mg/dL (n=28; 95% CI 188-157) than the mean preintervention PPBG of 206 mg/dL among nonresponders (n=23, nonresponders; 95% CI 234-178; *P*=.03; Figure 4). The responders reduced their PPBG by −32 mg/dL (n=21; 95% CI −16.4 to −47.2) as compared with 1.25 mg/dL (n=20; 95% CI 23.77 to −21.27; *P*=.03) among the nonresponders (Figure 4).

### Change in BMI and Weight

**Figure 5:**
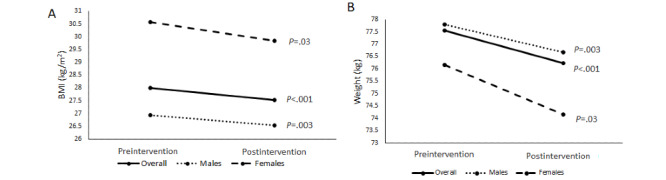
Change in BMI and weight. Difference between the (A) mean BMI and (B) mean weight reported in the first week and final week of the intervention.

There was a significant change in BMI (Figure 5) and in weight (Figure 5). The change in the mean BMI was −0.47 kg/m^2^ (n=59; 95% CI −0.22 to −0.71; *P*<.001), and the mean BMI reduction in males and females was −0.38 kg/m^2^ (n=42; 95% CI −0.14 to −0.62; *P*=.003) and −0.73 kg/m^2^ (n=17; 95% CI −0.12 to −1.34; *P*=.03), respectively. The change in mean weight was −1.32 kg (n=59; 95% CI −0.63 to −2.01; *P*<.001), and the mean weight change in males and females was −1.11 kg (n=42; 95% CI −0.40 to −1.83; *P*=.003) and −2.00 kg (n=17; 95% CI −0.38 to −3.62; *P*=.03), respectively.

The responders reduced their weight by −2.36 kg (n=27; 95% CI −1.32 to −3.41) as compared with −0.26 kg (n=23; 95% CI 0.42 to −0.95; *P*=.005) among the nonresponders. There was no significant difference in the mean preintervention weight between responders and nonresponders.

## Discussion

### Principal Findings

This study assessed the effectiveness of the Wellthy CARE digital therapeutics platform to improve glycemic control (reduction in HbA_1c_ levels and blood sugar levels) and reduce weight in patients of South Asian origin with T2D after a duration of 16 weeks of the program. Patients who used the digital therapeutic for 16 weeks reduced their HbA_1c_ levels by 0.49% (n=102), FBG by 11 mg/dL (n=51), PPBG by 21 mg/dL (n=51), weight by 1.32 kg (n=59), and BMI by 0.47 kg/m^2^ (n=59). Patients with the highest engagement significantly reduced their HbA_1c_ levels by 0.84%, FBG by 21.4 mg/dL, and PPBG by 22.03 mg/dL. There was a higher reduction in HbA_1c_, FBG, and PPBG levels as program engagement increased.

The effectiveness of the digital therapeutic was suggested by the significant reduction in HbA_1c_ levels. A 0.5% to 1% change in HbA_1c_ level is considered clinically significant to reduce the risk of comorbid conditions; even the US Food and Drug Administration requires a 0.4% change in HbA_1c_ level for drug evaluations [24]. The results of the United Kingdom Prospective Diabetes Study indicated that a 0.9% decrease in HbA_1c_ level was associated with a 25% reduction in microvascular complications, a 10% decrease in diabetes-related mortality, and a 6% reduction in all-cause mortality [25,26]. Hence, the average reduction of 1.34% in HbA_1c_ among the responders is significant in reducing the risk of complications and mortality.

FBG and PPBG levels are indicators of glycemic control and both are correlated with HbA_1c_ [27]. PPBG level has been shown to predict cardiovascular risk and all-cause mortality and has been shown to have a stronger correlation with HbA_1c_. There was a reduction in PPBG levels among responders by 32 mg/dL, which indicates an additional benefit of reduction in cardiovascular risk among these patients.

Weight reduction is one of the goals in the management of diabetes. Weight is associated with an increased risk of cardiovascular diseases [28], and a reduction in weight is associated with improvements in HbA_1c_ levels [29]. Although there was only a modest reduction of 1.32 kg weight among the participants after 16 weeks of the program, the significant reduction in weight among the responders (2.36 kg) highlights the importance of reducing weight in improving HbA_1c_ levels. It was also observed that females showed a higher reduction in mean BMI than males, even when no statistically significant difference between the BMI of the 2 groups was present at the preintervention stage. There could be several reasons for this, including differences in metabolism or hormonal balance between the 2 groups [30,31]. A substantial reason for such an effect could not be analyzed in this analysis.

To our knowledge, this work is one of the first to report the real-world effectiveness of implementing a digital therapeutic that is a structured, behavioral, and self-management program, which is augmented and delivered using a mobile-enabled app and an AI-powered decision support system for South Asian patients with T2D. Furthermore, the study highlighted the effect of the digital therapeutic to improve self-management of diabetes by demonstrating improvement in HbA_1c_, FBG, PPBG levels and reduction in BMI and weight. The real-world feasibility and acceptance were demonstrated by the observation that most of the users entered their HbA_1c_ readings during the program.

In recent years, considerable evidence has been generated about the effect of digital therapeutics in effectively promoting self-care and health behavior changes among patients with chronic diseases and mental health issues [32,33]. In countries such as India that lack appropriate diabetes self-management programs and where there is an overdependence for disease knowledge and support on physicians, the need for appropriate digital interventions is even more pronounced. Moreover, it has been reported that diabetes management interventions targeted at South Asian patients are heterogeneous, yielding variable and limited success in reducing HbA_1c_ levels [34]. Geographic and cultural diversity are significant barriers to the adoption of new treatments and technologies. However, the results here are a vindication of the value of a digital therapeutic such as Wellthy CARE across a wide range of population types and suggest that digital intervention provides a reasonable alternative in not only plugging some of the resourcing gaps in underserved countries but also has the potential to provide meaningful improvements in diabetes management.

### Limitations

One of the limitations of this study is the lack of a control group, as real-world data were retrospectively analyzed. Other limitations include selection bias due to multiple approaches (physician-recommended and voluntary approach) used for the selection of participants and reliance on self-reported disease biomarkers. The program was conducted for a short duration, and we did not independently quantify the effect of other behavioral and lifestyle measures on glycemic outcomes. This analysis can prove to be beneficial in providing important insights, especially when comparing the results of the Wellthy CARE platform with other digital therapeutics platforms. Real-world data helped in the study of people outside a controlled set of conditions. This increased the variability in the study; however, such studies have shown to be very helpful for the evaluation of new technologies. A variation in the number of male and female participants was noted, which was an undesirable bias due to social, economic, or other factors. The loss of data during follow-up also limited the scope of the study. However, the reporting for HbA_1c_ values was substantially high, with 62% of the enrolled users logging their preintervention and postintervention values during the entire program. This study showed significant improvement in HbA_1c_ and blood sugar values after a 16-week program, suggesting the potential of the Wellthy CARE platform for improving glycemic control in patients with T2D. However, future studies with a larger sample with better control will be able to further establish the effectiveness of the program.

### Strengths

The strength of this work was its design, which enabled us to closely simulate real-world implementations. The effectiveness of the intervention in documenting meaningful changes in HbA_1c_ levels, positive relationship of mobile app engagement with improvement in glycemic control, good rate of retention, and successful data collection contributed to its strength. The other strengths were that the app was implemented with the support of the same developmental processes and core support team that were involved in the development phase.

### Conclusions

This work is one of the first to report the real-world effectiveness of a digital therapeutic that was a structured, behavioral, and self-management intervention delivered using a mobile-enabled app and an AI-powered decision support system—Wellthy CARE in improving glycemic control among South Asians with T2D. The intervention demonstrated incremental reduction in HbA_1c_, FBG, and PPBG values with greater levels of engagement, suggesting the feasibility, acceptance, and value of using a digital therapeutic in resource-constrained countries of South Asia. The study findings can be explored further to evaluate the long-term acceptability, cost-effectiveness, and durability of the principal findings and its feasibility to be applied to a larger, culturally similar population.

## Appendix

Multimedia Appendix 1 Schematic of the Wellthy CARE digital therapeutics platform.

## Abbreviations

AI: artificial intelligence
FBG: fasting blood glucose
HbA_1c_: hemoglobin A_1c_
PPBG: postprandial blood glucose
T2D: type 2 diabetes
