# Supplementary figures and images for: Evaluating Glycemic Control in Patients of South Asian Origin With Type 2 Diabetes Using a Digital Therapeutic Platform: Analysis of Real-World Data
Source: J Med Internet Res. 2021 Mar 25;23(3):e17908. doi: 10.2196/17908 (PMC8074838; doi:10.2196/17908)

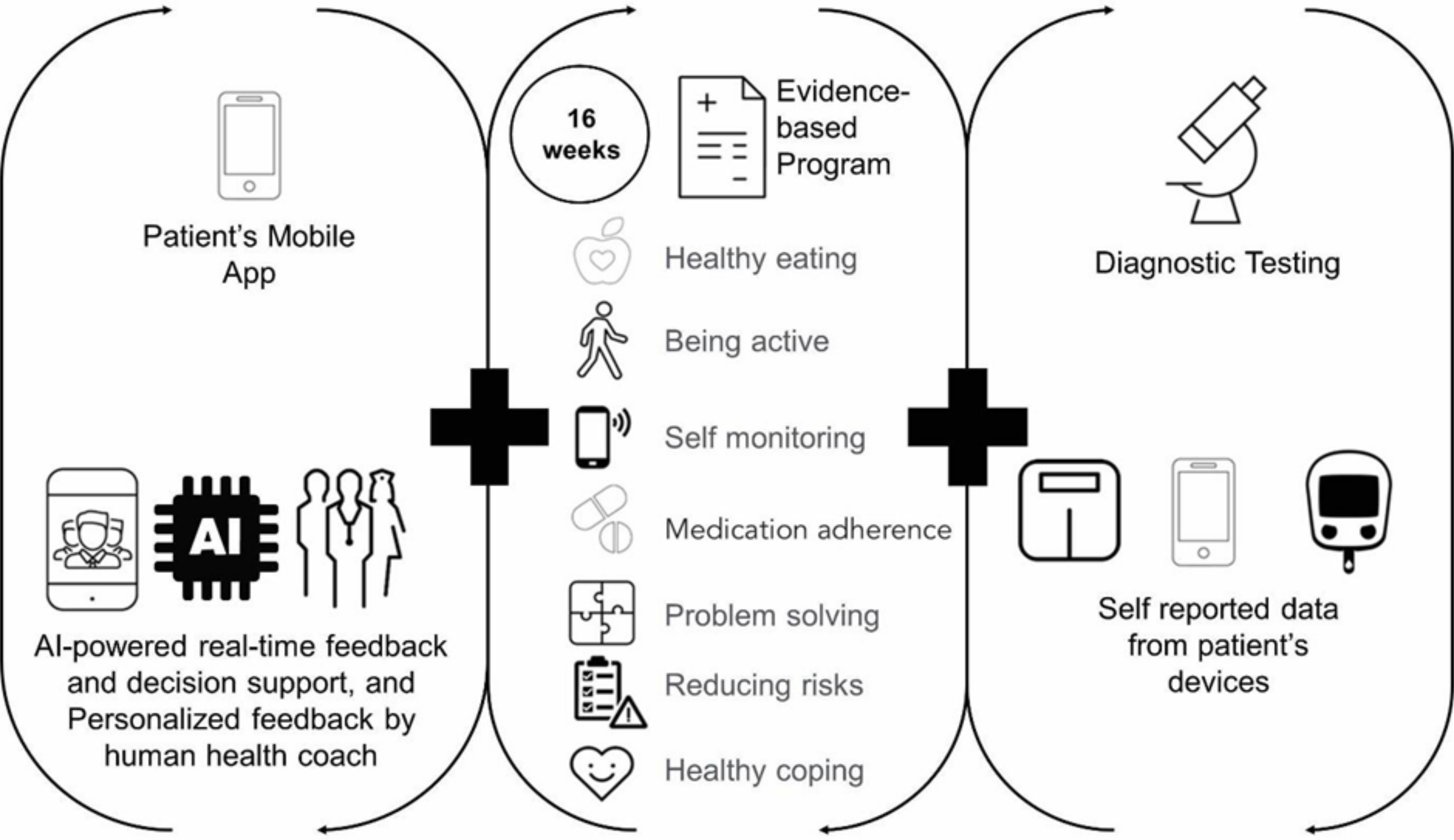

Supplement: Multimedia Appendix 1 [file jmir_v23i3e17908_app1.png]
